# Supplementary material for: Natural hybridization in heliconiine butterflies: the species boundary as a continuum
Source: BMC Evol Biol. 2007 Feb 23;7:28. doi: 10.1186/1471-2148-7-28 (PMC1821009; doi:10.1186/1471-2148-7-28)
Supplement: Additional File 1 — Hybrids between species of Heliconius and Eueides butterflies: a database. HTML file linking to database of all known wild-caught interspecific hybrid specimens in the Heliconiina, consisting of introductory text, a list of specimens, together with collection data and photographs of the specimens, and links to information about some artificial hybrids and mutants in the group. This is an edited copy of our online database of Heliconius hybrids [102]. To view database, download zip file and extract to a separate folder, then open index.html within that folder. [file 1471-2148-7-28-S1.zip › lybvib01.html]

hybrid lybvib01


---


Hybrid between *Eueides* *?vibilia unifasciatus* and *E. lybia lybia*
  
Brazil
  
© K. Brown Jr.

Return to table of hybrids

To next hybrid  
To previous hybrid (NONE!)

```
NOTES

No:                      1
Genus of species 1:      Eueides
Species 1:               ?vibilia
Subspecies of species 1: unifasciatus
Genus of species 2:      Eueides
Species 2:               lybia
Subspecies of species 2: lybia
Sex:                     m
Country:                 Brazil
Locality:                Benjamin Constant
Year:
Photo no.:               lybvib01
Named hybrid:
Collection:              MN/UFRJ
Collector:               K. Brown photo: MN4
Author/publication:
Notes:                   F1; or maybe F1 lybia x tales, lybia x isabella, lybia x heliconioides?
```

**Last updated:** 18 October 2003

---
